# Preoperative exercise and recovery after cardiac surgery: a meta-analysis

**DOI:** 10.1186/s12872-019-01308-z

**Published:** 2020-01-08

**Authors:** Yu-Ting Zheng, Jiang-Xu Zhang

**Affiliations:** 1 Department of Interventional vascular surgery, The Fourth Affiliated Hospital of Harbin Medical University, Harbin, China; 2 Minimally Invasive Tumor Therapies Center, Beijing Hospital, No. 1 Dongdan Dahua Road, Dongcheng District, Beijing, 100730 China

**Keywords:** Preoperative exercise, Cardiac surgery, Meta

## Abstract

**Background:**

To evaluate the association between preoperative exercise and recovery after cardiac surgery.

**Methods:**

Literature comparing preoperative exercise and the control group for patients receiving cardiac surgery was retrieved in multiple databases. Review Manager 5.2 was adopted for meta-analysis, sensitivity analysis and bias analysis.

**Results:**

Finally, 6 relevant studies satisfied the inclusion criteria. There was significant difference in length of stay in intensive care unit (ICU) (MD- = 1.35, 95%CI [− 2.64, − 0.06], *P* = 0.04; *P* for heterogeneity < 0.0001, *I*^*2*^ = 88%) and physical function after operation (*P* of heterogeneity = 0.32, *I*^*2*^ = 12%, Z = 9.92, *P* of over effect< 0.00001). The meta-analysis suggested that there was no significant difference in white blood cell count (WBC) at postoperative day 7 and mental health after operation between the exercise group and the control group. Limited publication bias was observed in this study.

**Conclusion:**

Preoperative exercise including inhaled muscle training, aerobics, resistance training and stretching could promote recovery after cardiac surgery.

## Background

Despite the rapid changes in science and technology, cardiac surgery is still accompanied by complications that increase morbidity and mortality [1, 2]. Postoperative pulmonary complications (PPC) are a serious problem after cardiac surgery, and the reported prevalence is between 5 and 90%, depending on the definition of complications. These complications can lead to adverse consequences and prolonged hospital stays, leading to increased hospital costs and an important cause of hospital mortality [3, 4].

Preoperative exercise training can prevent postoperative pulmonary complications [3–5]. Several reports have shown that interventional therapy can reduce the incidence of postoperative complications and postoperative hospital stay in patients undergoing cardiac surgery [3–7].

It has been reported that the incidence of postoperative pulmonary complications is closely related to the status of preoperative pulmonary function, so by improving preoperative pulmonary function, postoperative pulmonary complications can be effectively prevented [8]. Respiratory muscles, like skeletal muscles, can improve their muscle strength and endurance through exercise. Two weeks of deep inspiratory respiratory muscle exercise can effectively improve blood perfusion of respiratory muscle, enhance the quality and intensity of muscle fibers, effectively improve the endurance of respiratory muscle, and at the same time increase the number of alveoli involved in gas exchange [9–11]. This not only improves the ability of lung ventilation, but also improves the ability of lung ventilation, thus increasing the reserve of respiratory function and the ability of respiratory muscle to resist fatigue.

Several articles have analyzed association between preoperative exercise and recovery after cardiac surgery, in which there exist various research designs, recruit, exclusion criteria and methods. A meta-analysis was needed to analyze the relationship between preoperative exercise and recovery after cardiac surgery.

## Methods

### Literature search strategy

Between January 2001 and March 2019, a number of electronic databases were used, including PubMed, Ovid EMBASE, Ovid MEDLINE and Cochrane databases, and China National Knowledge Infrastructure (CNKI). Search for keywords: exercise or exercise planning and heart surgery. There are no restrictions on publishing languages. The study was initially reviewed for title and abstract. In addition, it is checked whether the referenced catalog of all the retrieved papers contains qualified articles.

### Study selection

Studies were included if:

- They were considered as randomized trials or case-control studies.
- They compared exercise versus controls.
- They involved patients receiving cardiac surgery.

Studies were excluded if:

- They were case studies/meta-analyses/letter to editors.
- Comparison between exercise and controls was not made.
- Patients received other surgery.
- Data in research was limited or insufficient.
- They were duplicates.

### Data extraction and quality assessment

Two reviewers (Zheng and Zhang) independently scanned the full text of the manuscript and extracted the following data from each eligible article: first author’s name, age and gender distribution of the patient, country, year of publication, sample size and time on collection of the article. The methodological quality of the study was evaluated by the Cochrane bias assessment tool [12].

### Statistical analysis

The analysis used Review Manager 5.2 (Cochrane Collaboration, Oxford, UK). The effect size of the numerical variables is described as the mean difference with 95% confidence interval (CI); the classification data is expressed as RR with 95%CI. We tested the heterogeneity between studies using the I^2^ metric. In our study, 25% (I^2^ = 25), 50% (I^2^ = 50) and 75% (I^2^ = 75) were considered low, medium and high heterogeneity, respectively. When I^2^ is greater than 50%, a random effects model is used; otherwise, a fixed effect model is employed [12].

## Results

### Search process

**Fig. 1:**
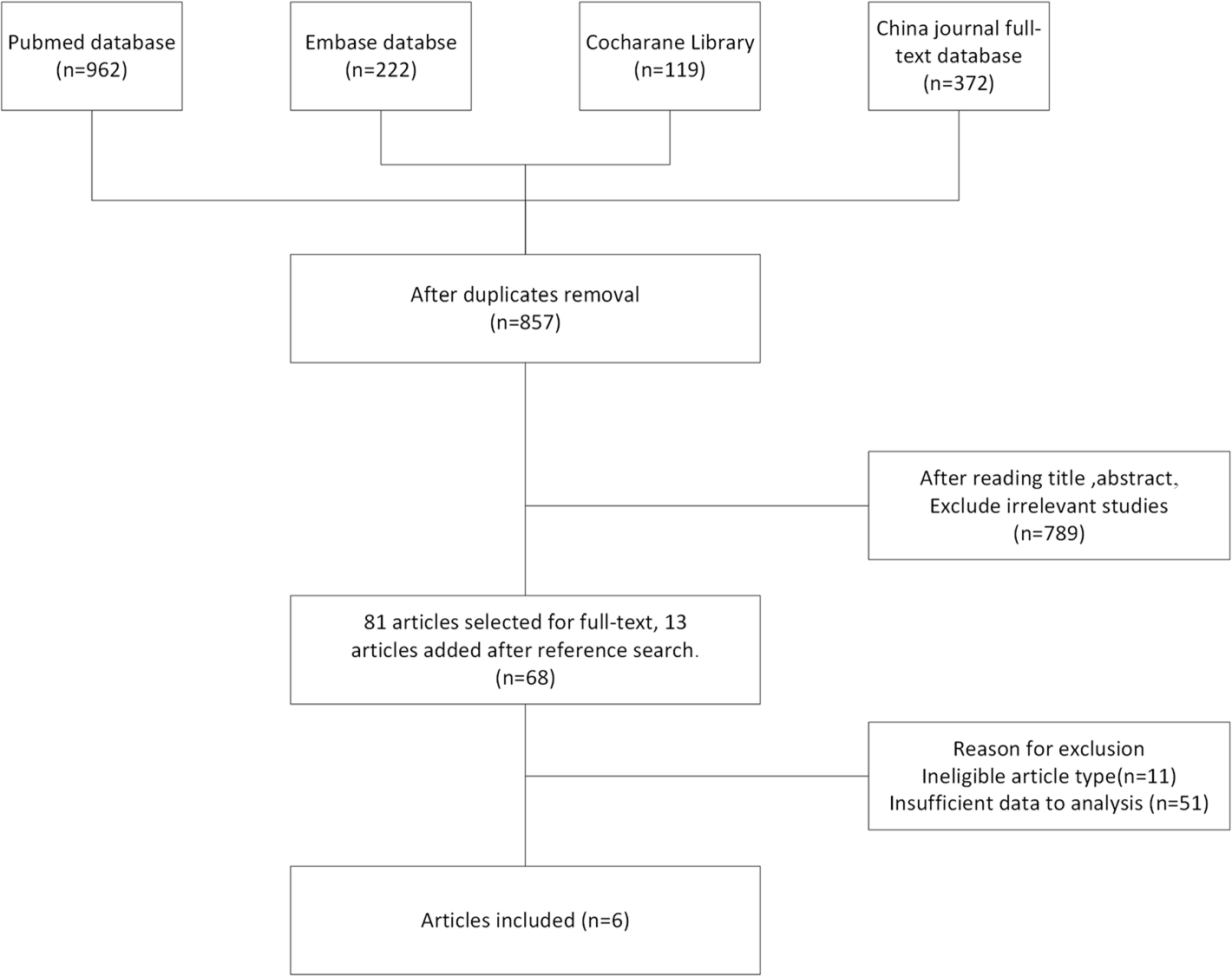
The flow diagram of the study selection

A total of 857 articles on patients receiving exercise program and controls were initially identified. After removal of duplicated publications, 6 articles eventually met the inclusion criteria. Totally 851 articles were excluded for duplication, irrelevant studies, inappropriate data, reviews, lack of or inappropriate control, other detection methods, and no full-text articles available. Figure 1 shows the search process and the reasons for exclusion. Among these 6 articles, 4 were involved in length of stay in the ICU, 3 were involved in abnormal WBC at postoperative day 7, 3 were involved in postoperative physical function, and 3 were involved in postoperative mental health. The process of searching articles and writing paper follows the PRISMA approach [11].

### Characteristics of the included studies

Table 1 lists the characteristics of the included studies, including first author, year of publication, country, age range, gender distribution (male/female), sample size, and time of recruitment. All of these articles were published between 2001 and 2019. Sample sizes range from 35 to 346. A total of 752 patients were enrolled in these studies, with 275 and 477 in the exercise and control groups, respectively. Among included studies, exercise programs include inhaled muscle training, aerobics, resistance training and stretching [13–18].

**Table 1:** Characteristics of studies included in the meta-analysis

Study	Year	Nation	Age(years)	Gender(male/female)	groups	n	Recruitment time
Chen [13]	2019	China	61.7 ± 7.9	141/56	Exercise	98	June 2018 to September 2018
					Control	99	
Cho [14]	2014	Japan	64.5 ± 11.7	69/3	Exercise	18	February 2007 to January 2013
					Control	54	
Shi [15]	2008	China	57.3 ± 12.3	38/25	Exercise	35	September 2005 to May 2006
					Control	28	
Timmerman [16]	2010	Netherlands	61.2 ± 12.7	29/10	Exercise	15	January 2006 to June 2006
					Control	24	
Tung [17]	2012	China	53.2 ± 10.7	28/7	Exercise	15	September 2010 to April 2011
					Control	20	
Valkenet [18]	2013	Netherlands	67.3 ± 11.2	230/116	Exercise	94	January 2008 to December 2009
					Control	252	

### Results of quality assessment

**Fig. 2:**
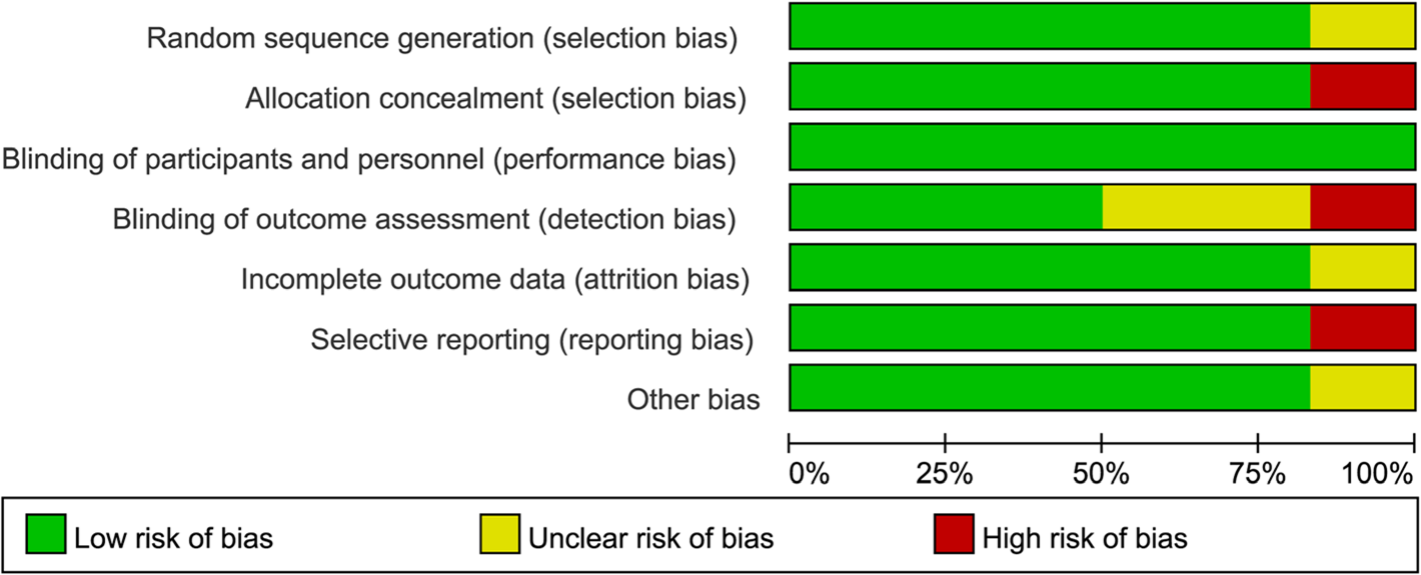
Assessment of the quality of the included studies: low risk of bias (green hexagons), unclear risk of bias (yellow hexagons), and high risk of bias (red hexagons)

**Fig. 3:**
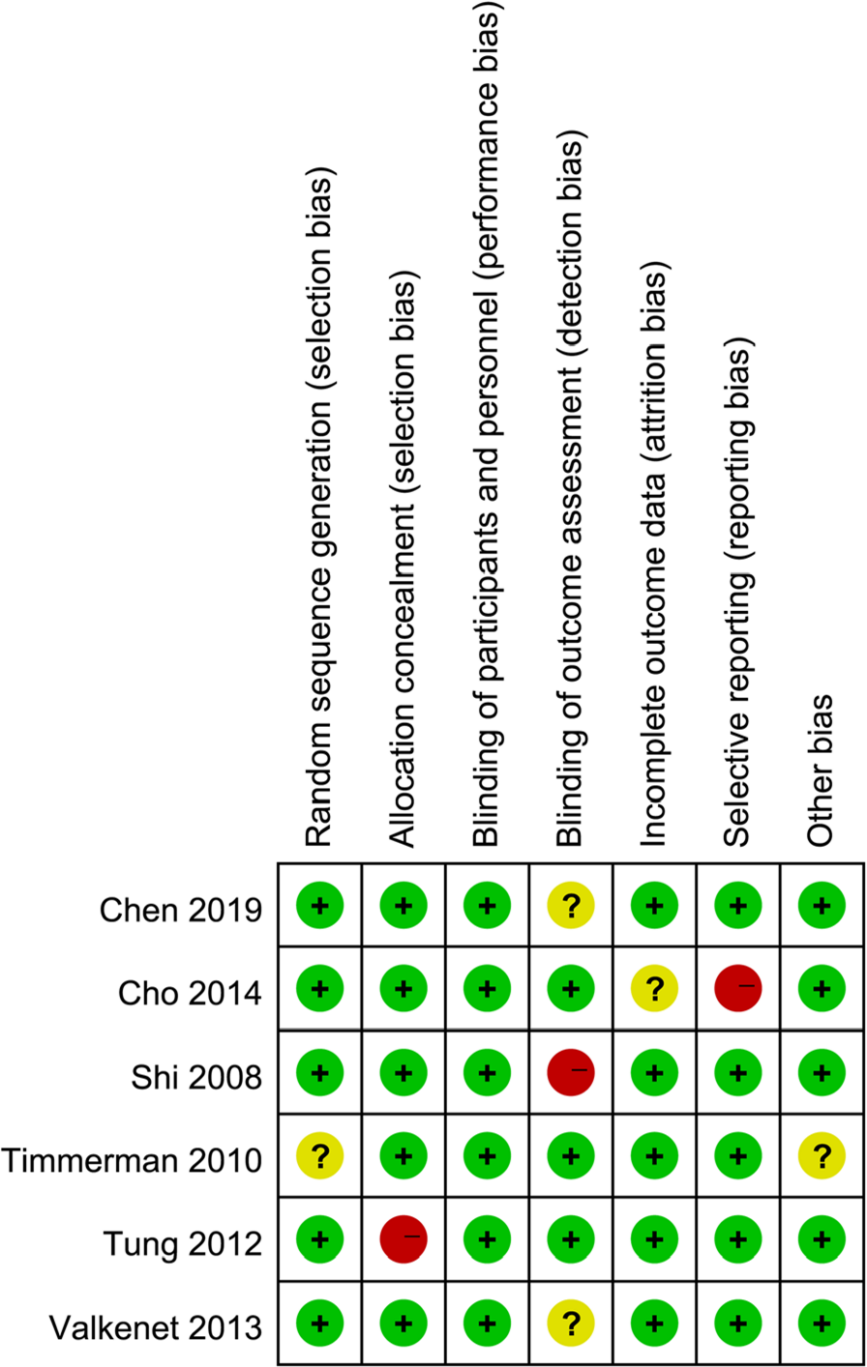
Quality assessment of the included studies

The Cochrane bias assessment tool was used to evaluate the risk of bias in the 6 trials. One trial showed selection bias, 1 trial showed detection bias, and 1 trial showed reporting bias. The detailed results of the quality assessment are listed in Fig. 2 and Fig. 3.

### Results of heterogeneity test

#### Meta-analysis about length of stay in ICU

**Fig. 4:**
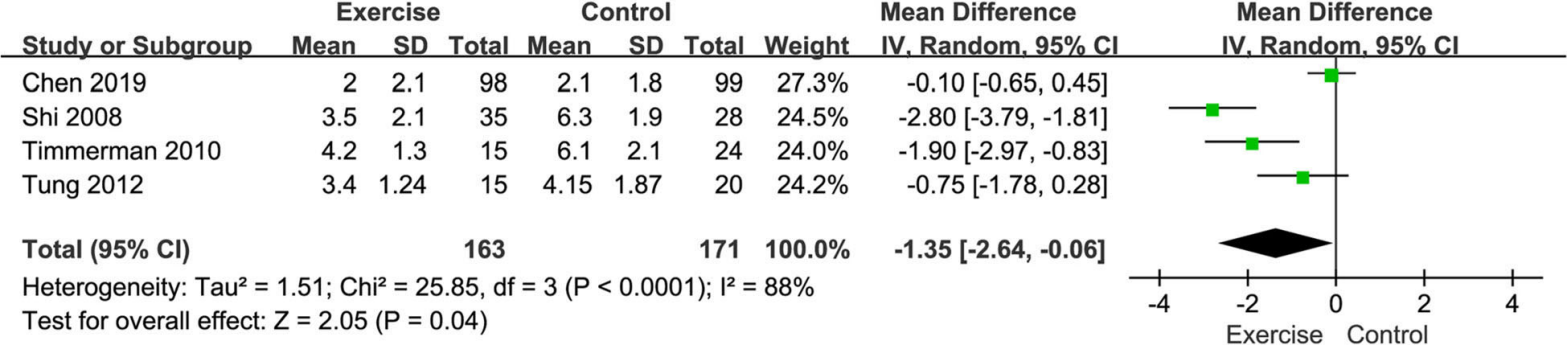
Forest plots of length of stay in the ICU

Four of the 6 included trials studied length of stay in the ICU in the exercise group and the control group (Fig. 4). The combined result suggested that there was significant difference in length of stay in the ICU between the exercise group and the control group. The control group had significantly longer stay of ICU than the exercise group (MD- = 1.35, 95%CI [− 2.64, − 0.06], *P* = 0.04; *P* for Heterogeneity < 0.0001, *I*^*2*^ = 88%).

#### Meta-analysis about abnormal WBC at postoperative day 7

**Fig. 5:**
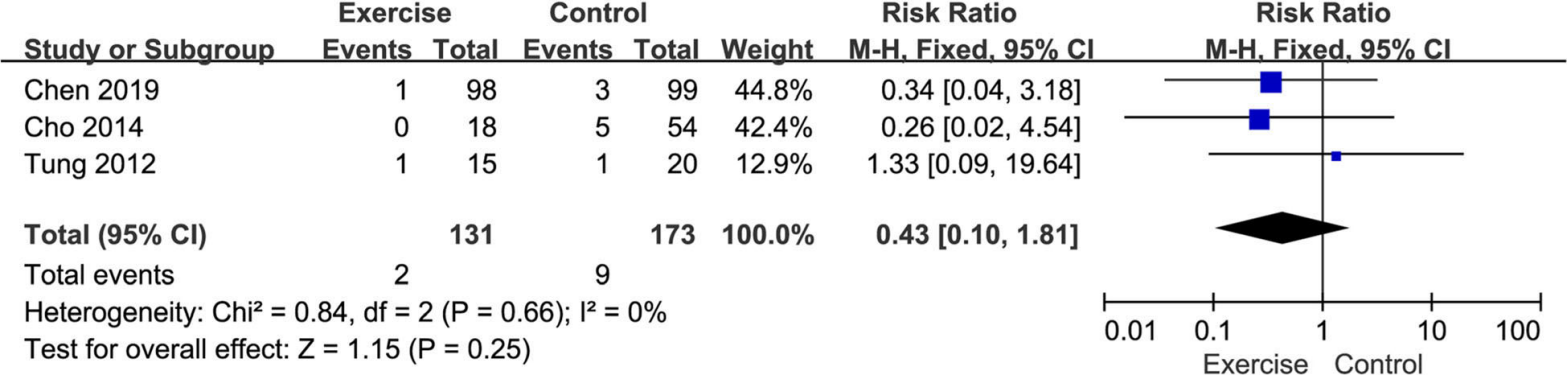
Forest plots of postoperative abnormal WBC at postoperative day 7

Three included trials studied abnormal WBC at postoperative day 7 between the exercise group and the control group. As shown in the forest plot (Fig. 5), the result of the meta-analysis showed no significant statistical difference in abnormal WBC at postoperative day 7 between the exercise group and the control group (RR = 0.43, 95%CI [0.10, 1.81], *P* = 0.25; *P* for heterogeneity = 0.66, *I*^*2*^ = 0%).

#### Meta-analysis of postoperative physical function

**Fig. 6:**
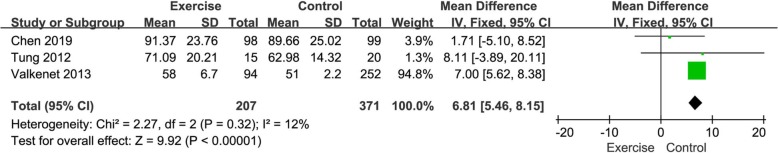
Forest plots of postoperative physical function

In the analysis of postoperative physical function in the exercise group and the control group, 3 articles were included. The results of heterogeneity test showed that fixed effect model wad was needed to analyze the data (*P* of heterogeneity = 0.32, *I*^*2*^ = 12%). The overall effect was significant and the overall MD was 6.81 (Z = 9.92, *P* of over effect< 0.00001), which showed that the exercise group had better postoperative physical function than the control group (Fig. 6).

#### Meta-analysis of postoperative mental health

**Fig. 7:**
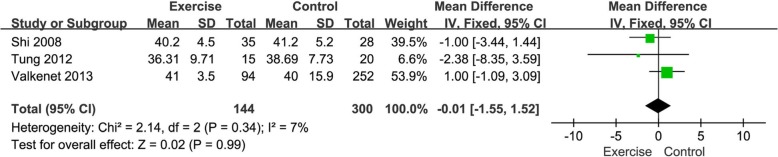
Forest plots of postoperative mental health

Three of the 6 studies involved postoperative mental health. Three articles showed no difference in postoperative mental health between the exercise group and the control group. The results of the meta-analysis indicated that postoperative mental health in exercise subjects was similar to controls (MD = -0.01, 95%CI [− 1.55, 1.52], *P* = 0.99; *P* for Heterogeneity = 0.34, *I*^*2*^ = 7%). The forest plot for postoperative mental health in the exercise group and the control group is shown in Fig. 7.

### Results of sensitivity analysis and publication Bias

**Fig. 8:**
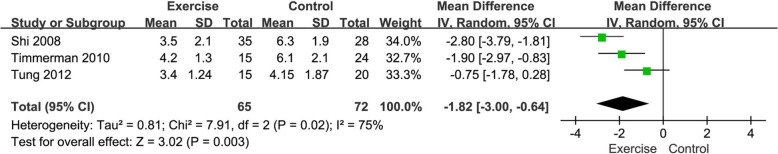
**a** Sensitivity analysis forest plots about length of stay in ICU excluding apparent outlier study (Chen et al. [13]) **b** Funnel plot of publication bias

To examine the stability of the outcome, a sensitivity analysis was needed. A relative outlier was excluded, and the results demonstrated that in heterogeneity, *I*^*2*^ of sensitivity for exercise and control value changed from 88 to 75%. It indicated that the heterogeneity was mainly due to the study by Chen et al. in 2019. The forest plot without Chen et al.’s article is shown in Fig. 8a.

A funnel plot for length of stay in the ICU using exercise and control was performed. All the studies were included in the plot. To some extent, the results indicated that there existed some publication bias since the symmetrical characteristic of the funnel plot was not so good (Fig. 8b).

## Discussion

The World Health Organization (WHO) said that by 2020, heart disease will be the world’s leading cause of death, and it is estimated that 25 million people will suffer from heart disease each year [19]. In the 1940s, researchers put forward active exercise for heart disease. People gradually realized the benefits of active exercise to the cardiac system. Especially in the treatment of cardiac disease, more and more attention has been paid to active exercise. Studies have shown that if patients with heart disease can carry out regular physical activity and understand how to control cardiac risk factors and postoperative events, rehospitalization rate and mortality rate can be significantly reduced. In recent decades, for patients with heart disease, in addition to drug treatment and lifestyle changes, active exercise has become an important treatment [19–21].

Insufficient exercise training for patients with heart disease may lead to adverse consequences and increase surgical complications [22, 23]. Preoperative active exercise among patients with coronary heart disease can promote their own physiological changes, thus promoting recovery after CABG. Physical activity increases the cardiac reserve of patients, reduces oxygen consumption of the myocardium, reduces the load of contraction and relaxation, and improves the metabolism of blood lipids and carbon oxides. Active exercise can effectively improve and correct the imbalance of coronary artery endothelial function, and then promote the rehabilitation process of patients with coronary heart disease [17, 24].

Some studies have shown that exercise therapy can improve the range of motion in the joint, enhance muscle tension, promote blood circulation, regulate the function of the main organs of the body, and improve the mood and the ability of daily life [25, 26]. Exercise can improve cerebral blood flow, increase heart rate, increase ventilation and promote recovery. According to the results of length of stay in ICU, this study shows that active preoperative physical activity can shorten postoperative hospital stay and reduce postoperative complications in patients with CABG.

Our results support the point that preoperative exercise can shorten the stay in ICU among patients receiving cardiac surgery, and this conclusion is consistent with Valkenet et al. who showed that prophylactic physiotherapy of inspiratory muscle training for 5 days before cardiac surgery increased inspiratory intensity, reduced the incidence of postoperative pulmonary complications and shortened hospital stay [24].

The postoperative physical function in the exercise group was better than that of the control group, and the reason might be that preoperative inspiratory muscle training is a useful intervention for patients undergoing cardiac surgery. Inspiratory muscle training improves forced inspiratory volume, forced vital capacity and maximum voluntary ventilation in the first second of cardiac surgery, which may improve their ability to cough, discharge secretions, and reduce postoperative pulmonary complications.

In the analysis of postoperative abnormal WBC at postoperative day 7 and postoperative mental health, there was no significant difference between the exercise group and the control group in our study. These might indicate that the postoperative complications and mental health had no difference whether patients receive preoperative exercise.

## Conclusions

In conclusion, the present study showed that length of stay in the ICU and postoperative physical function in the exercise group were better than those of the control group. But no significant difference was observed in complications and mental health. Our results suggested that preoperative exercise may be helpful for postoperative cardiac recovery. However, our findings should be carefully considered with caution due to small sample size. Studies in various areas with large study populations are essential to further confirm our findings in the future.

## Data Availability

The datasets generated and analyzed during the current study are available from the corresponding author on reasonable request.

## Abbreviations

CI: Confidence interval
ICU: Intensive care unit
PPC: Postoperative pulmonary complications
WBC: White blood cell count
WHO: World Health Organization

## References

[1] Beaupre LA (2004). The effect of a preoperative exercise and education program on functional recovery, health related quality of life, and health service utilization following primary total knee arthroplasty. J Rheumatol.

[2] Cabilan CJ, Hines S, Munday J (2015). The effectiveness of prehabilitation or preoperative exercise for surgical patients: a systematic review. JBI Database System Rev Implement Rep.

[3] Kalogianni A (2016). Can nurse-led preoperative education reduce anxiety and postoperative complications of patients undergoing cardiac surgery?. Eur J Cardiovasc Nurs.

[4] Karenovics Wolfram, Licker Marc, Ellenberger Christoph, Christodoulou Michel, Diaper John, Bhatia Chetna, Robert John, Bridevaux Pierre-Olivier, Triponez Frédéric (2017). Short-term preoperative exercise therapy does not improve long-term outcome after lung cancer surgery: a randomized controlled study†. European Journal of Cardio-Thoracic Surgery.

[5] Karenovics W (2016). B-004DOES SHORT-term preoperative exercise therapy influence long-term lung functional outcome following lung cancer surgery?. Interactive Cardiovasc Thoracic Surg.

[6] Fontes Cerqueira TC (2018). Ambulation capacity and functional outcome in patients undergoing neuromuscular electrical stimulation after cardiac valve surgery. Medicine.

[7] Hirschhorn AD (2012). Does the mode of exercise influence recovery of functional capacity in the early postoperative period after coronary artery bypass graft surgery? A randomized controlled trial. Interact Cardiovasc Thorac Surg.

[8] Kose EA (2012). Preoperative exercise heart rate recovery predicts intraoperative hypotension in patients undergoing noncardiac surgery. J Clin Anesth.

[9] Lemanu DP (2013). Effect of preoperative exercise on cardiorespiratory function and recovery after surgery: a systematic review. World J Surg.

[10] Mcglade DP, Poon AB, Davies MJ (2001). The use of a questionnaire and simple exercise test in the preoperative assessment of vascular surgery patients. Anaesth Intensive Care.

[11] Moher D, Liberati A, Tetzlaff J (2009). Preferred reporting items for systematic reviews and meta-analyses: the PRISMA statement. Revista Española De Nutrición Humana Y Dietética.

[12] Higgins JP, Altman DG, Gøtzsche PC, Jüni P, Moher D, Oxman AD, Savović J, Schulz KF, Weeks L, Sterne JA (2011). The Cochrane Collaboration’s tool for assessing risk of bias in randomised trials. BMJ.

[13] Chen X (2019). The effects of five days of intensive preoperative inspiratory muscle training on postoperative complications and outcome in patients having cardiac surgery: a randomized controlled trial. Clin Rehabil.

[14] Cho H (2014). Matched pair analysis to examine the effects of a planned preoperative exercise program in early gastric Cancer patients with metabolic syndrome to reduce operative risk: the adjuvant exercise for general elective surgery (AEGES) study group. Ann Surg Oncol.

[15] Ft S (2008). Influence of preoperative physicalactivity on the prognosis of patients after coronary artery bypass surgery. J Xinxiang Med College.

[16] Timmerman H (2010). Feasibility and preliminary effectiveness of preoperative therapeutic exercise in patients with cancer: a pragmatic study. Physiotherapy Theory Practice.

[17] Tung H (2012). Effects of a preoperative individualized exercise program on selected recovery variables for cardiac surgery patients: a pilot study. J Saudi Heart Assoc.

[18] Valkenet K (2013). Effect of Inspiratory Muscle Training Before Cardiac Surgery in Routine Care. Physical Therapy.

[19] Sebio GR (2016). Functional and postoperative outcomes after preoperative exercise training in patients with lung cancer: a systematic review and meta-analysis. Interact Cardiovasc Thorac Surg.

[20] Sebio GR (2017). Preoperative exercise training prevents functional decline after lung resection surgery: a randomized, single-blind controlled trial. Clin Rehabil.

[21] Shoyeb A (2010). Preoperative exercise echocardiography and perioperative cardiovascular outcomes in elderly patients undergoing cancer surgery. Am J Geriatric Cardiol.

[22] Supino PG (2013). Usefulness of preoperative exercise tolerance to predict late survival and symptom persistence after surgery for chronic nonischemic mitral regurgitation. Am J Cardiol.

[23] Supino P (2010). Preoperative exercise capacity best predicts survival after surgery for chronic nonischemic mitral regurgitation. J Am College Cardiol.

[24] Valkenet K (2011). The effects of preoperative exercise therapy on postoperative outcome: a systematic review. Clin Rehabil.

[25] Weinstein H (2007). Influence of preoperative exercise capacity on length of stay after thoracic cancer surgery. Ann Thorac Surg.

[26] Yang A, Sokolof J, Gulati A. The effect of preoperative exercise on upper extremity recovery following breast cancer surgery: a systematic review. Int J Rehabil Res. 2018. http://europepmc.org/article/MED/29683834. 10.1097/MRR.0000000000000288 29683834

